# Association Between Physical Activity Level and Training Volume in Adolescent Athletes

**DOI:** 10.3390/sports13060178

**Published:** 2025-06-06

**Authors:** Sebastian Sitko, Alejandro Legaz-Arrese, Joaquín Reverter-Masia, Diego Moliner-Urdiales, Vicenç Hernández-González, Saül Aixa-Requena, Enric Conesa-Milian, Carmen Mayolas-Pi

**Affiliations:** 1 Faculty of Health and Sport Sciences, University of Zaragoza, 50009 Zaragoza, Spain; sitko@unizar.es (S.S.); alegaz@unizar.es (A.L.-A.); carmayo@unizar.es (C.M.-P.); 2 Human Movement Sports Research Group, University of Zaragoza, 50009 Zaragoza, Spain; 3 Faculty of Education, Psychology and Social Work, University of Lleida, 25003 Lleida, Spain; joaquim.reverter@udl.cat (J.R.-M.); sar.lete35@gmail.com (S.A.-R.); enric.conesa@udl.cat (E.C.-M.); 4 Human Movement Research Group (RGHM), University of Lleida, Plaça de Víctor Siurana, 25003 Lleida, Spain; 5 LIFE Research Group, University Jaume I, 12071 Castellón, Spain; dmoliner@uji.es

**Keywords:** inactivity, practitioners, sport, competitive athletes, competitive level

## Abstract

Organized sports are assumed to boost overall physical activity, but evidence suggests structured training does not always increase general activity outside formal sessions. This study explores the link between physical activity levels and weekly training hours in adolescent athletes across sports and competition levels. A total of 10,196 participants aged 11 to 19 were included in the analyses. Participants were classified into seven groups: inactive, somewhat active, non-competitive athletes, and athletes competing at the local, regional, national, or international level. They completed the Spanish version of the Physical Activity Questionnaire and answered questions regarding their performance level, training volume, and socioeconomic status. Girls exhibited lower physical activity levels than boys, even at higher competition levels where both sexes had comparable training hours. Competitive athletes reported the highest physical activity levels. In competitive athletes, physical activity levels decreased with age despite a substantial increase in weekly training hours. They were similar across athletes competing in different sports and at different levels, despite significant differences in training hours. Many athletes, including those at the national and international levels, displayed low physical activity levels, and no clear relationship was found between physical activity level and weekly training hours. The current study provides valuable insights into adolescent physical activity patterns by sex, age, sport, and competition level. Girls showed lower physical activity than boys, even with similar training hours. Physical activity levels declined with age despite increasing training volumes, and no strong association was observed between physical activity and weekly training hours. These results reveal a discrepancy between structured training and overall activity levels, challenging assumptions about the impact of sports participation on daily physical activity.

## 1. Introduction

Physical activity is widely recognized as a fundamental component of adolescent health, playing a crucial role in physical, mental, and social development [1]. Regular engagement in physical activity has been linked to numerous benefits, including improved cardiovascular health, enhanced muscular fitness, better bone density, and a reduced risk of obesity and metabolic disorders [2]. Additionally, participation in sports has been associated with psychosocial advantages, such as improved self-esteem, greater social integration, and the development of discipline and teamwork skills [3]. However, despite these well-established benefits, research suggests that a significant proportion of adolescents fail to meet recommended physical activity guidelines, raising concerns about long-term health implications [4].

Participation in organized sports is often assumed to contribute positively to overall physical activity levels [5]. However, there is growing evidence that structured training sessions do not always translate into higher general activity levels outside of formal training environments [6]. Athletes may compensate for intensive training by reducing non-sport-related physical activity, leading to an overall activity pattern that differs from that of non-athletes [7]. Additionally, variations in training load, competition schedules, and sport-specific demands may result in disparities in total physical activity between athletes in different disciplines [8]. Understanding these relationships is crucial for designing training programs that optimize athletic development while ensuring adequate physical activity engagement.

Another critical factor influencing physical activity levels in adolescents is sex-related differences. Prior studies consistently report that girls tend to be less active than boys, a trend observed across both general populations and athletic cohorts. While differences in biological and psychosocial factors may contribute to this disparity, the extent to which training volume influences these differences remains unclear [9]. Furthermore, research has shown that physical activity levels tend to decline with age during adolescence, despite potential increases in training hours among competitive athletes [10]. The interplay between age, training load, and overall activity warrants further investigation to better understand the long-term implications of structured sports participation on youth development [11].

With over 25.000 entries in Pubmed, the Physical Activity Questionnaire (PAQ) is one of the most used self-reported tools to assess physical activity levels in adolescent populations. It provides a general overview of activity levels by categorizing various types and intensities of physical activity. However, its performance, when compared to objective methods, to discriminate sport-specific versus general physical activity levels remains unknown [12,13].

Given all the above, the present study aims to examine whether physical activity levels assessed by the PAQ are different between athletes and non-athletes, as well as to establish the relationship between physical activity levels and weekly training hours in adolescent athletes participating in different sports and competitive levels. Specifically, it seeks to explore differences by sex, age, sport type, and competition level to better understand how structured training influences overall activity patterns covered by this questionnaire.

## 2. Materials and Methods

### 2.1. Ethical Requirements

This cross-sectional study was based on self-reported data and conducted in accordance with the principles of the Declaration of Helsinki. The procedures of this project have been approved by the Ethical Committee for Clinical Research of the Sports Administration of Catalonia (30/CEICGC/2020).

### 2.2. Participants and Design

A total of 10,196 participants aged 11 to 19 completed the initial assessment, providing valid data on physical activity. Participants were classified into seven groups: inactive, somewhat active, non-competitive athletes, and athletes competing at the local, regional, national, and international levels. National and international athletes were recruited through Spanish and Regional Sports Federations, High-Performance Sports Centers, Sports Technification Centers, and the top 20 clubs of each sport discipline, based on performance level and sex. Recruitment was conducted via email, letter, and phone, including an introduction to the study, an explanation of its anonymous nature, and a link to the online questionnaire.

To be classified as an athlete in this study, participants had to report training at least two days per week. Competitive athletes were additionally required to have at least six months of continuous training and competition experience in a sport included in the Summer Olympic Games program. Participants who reported lower training frequencies were not excluded; instead, they were classified as inactive or somewhat active, based on their self-reported physical activity levels. To recruit inactive, somewhat active, non-competitive athletes, as well as locally and regionally competitive athletes, all students from secondary education centers in three representative Spanish provinces were invited to participate. Inactive subjects reported no engagement in sports and were categorized as having low physical activity levels according to the Patient-Centered Assessment and Counseling for Exercise (PACE) criteria, which define low activity as engaging in at least 60 min of moderate-to-vigorous physical activity for 0 to 2 days per week [14]. Somewhat active participants reported irregular sports participation and moderate physical activity levels, defined as engaging in at least 60 min of moderate-to-vigorous physical activity for 2 to 3.5 days per week. Non-competitive athletes reported engaging in sports at least twice per week without participating in competitions.

Exclusion criteria for all participants included the following: (1) chronic illness or any physical or psychological condition that could limit physical activity levels; (2) the presence of an injury that could affect participation in their respective sports or any variable considered in this study. Data collection was conducted from January to March to ensure that all study variables were assessed while the athletes were in an advanced phase of their training season, controlling for potential seasonal effects. Prior to data collection, informed consent was obtained from all participants and their parents or legal guardians, in accordance with ethical requirements. Table 1 presents the main characteristics of the participants, categorized by gender and performance level.

#### 2.2.1. Physical Activity and Sport

Participants completed the Spanish version of the Physical Activity Questionnaire for Children and Adolescents (PAQ-C and PAQ-A) [15]. They assessed their physical activity levels during leisure time, physical education classes, and various periods (lunchtime, afternoon, and evening) on school days and weekends over the past seven days. The PAQ-C consists of nine items, while the PAQ-A consists of eight items, both rated on a 5-point Likert scale. These scores were averaged to provide an overall physical activity score ranging from 1 to 5, with lower scores indicating lower physical activity levels.

Physical activity was also measured using a modified version of the PACE questionnaire for adolescents [16], adapted for epidemiological studies with European adolescents. This version asks participants about the number of days they accumulated at least 60 min of moderate-to-vigorous physical activity in the past 14 days [14]. Additionally, participants were asked to answer a set of independent questions regarding their sport participation. These included items on whether they had trained at least twice per week in the past six months, their primary sport, years of competitive experience, and their current number of training sessions and hours per week. Questions regarding performance level, season goals, and competitive classification were also included. Participants who competed in the highest national competitive category for their age, sex, and sport were classified as national-level athletes.

A traditional classification was adapted to differentiate between technical, power-based, gymnastic, combat, racket, team, and endurance sports [17]. Sports were coded as either team or individual based on whether they involved three or more athletes competing simultaneously [18].

#### 2.2.2. Socioeconomic Status

Subjective socioeconomic status was assessed with the question: “How wealthy do you consider your family to be”? Responses were classified into four categories: 1 (poor), 2 (not very poor), 3 (average), and 4 (rich or very rich) [19].

#### 2.2.3. Physical Evaluation

Body mass index (BMI) was calculated from self-reported weight and height [20]. Pubertal status was assessed using the Pubertal Development Scale [21], which has demonstrated acceptable validity and reliability [22]. For girls, the questionnaire addressed body hair, growth spurts, skin changes, menarche, and breast development. For boys, it included body hair, growth spurts, skin changes, facial hair, and voice changes. A five-level categorical scale was used, comparable to Tanner’s pubertal development stages [23], which classify secondary sexual characteristics into five distinct stages (from stage I: prepubertal to stage V: full physical maturity). This allows for standardized assessment of pubertal status in line with established biological maturation models. Participants were also categorized according to age (11–13, 14–16, 17–19 years).

#### 2.2.4. Other Variables

Participants were categorized based on geographic region in Spain (north/south) and the population size of their place of residence: fewer than 1000 inhabitants; 1001 to 10,000 inhabitants; 10,001 to 100,000 inhabitants; 100,001 to 500,000 inhabitants; and more than 500,000 inhabitants.

### 2.3. Statistical Analysis

All analyses were conducted using IBM SPSS Statistics v.26 (IBM Corp., Armonk, NY, USA). Descriptive statistics included means and standard deviations for continuous variables and percentages for categorical variables. The normality of the dependent variable was tested using the Kolmogorov–Smirnov test and skewness coefficient examination.

To assess the influence of independent factors on physical activity levels and weekly training hours, generalized linear models (GLMs) were employed. Since the dependent variables did not follow a normal distribution, a Gamma GLM was used. The independent factors analyzed included gender, age group (11–13, 14–16, 17–19 years), maturation stage (stages 1–5), competition level (inactive, slightly active, non-competitive athletes, and athletes competing at local, regional, national, or international levels), sport type (technical, power-based, gymnastic, combat, racket, team, endurance), and competition format (individual vs. team). When necessary, post hoc analyses with Bonferroni adjustment were performed. Confounding variables positively associated with physical activity participation were included as covariates, such as pubertal status, socioeconomic status, place of residence, and municipality size. Interactions among independent variables were controlled by conducting a two-factor analysis with confounding variables. The Spearman correlation test was used to evaluate the association between physical activity level and training hours based on the type of sport, in order to determine whether a significant relationship exists between these variables. A significance level of *p* < 0.05 was considered statistically significant.

## 3. Results

Regardless of age, boys demonstrated significantly higher physical activity levels than girls at all competition levels, according to the PAQ (*p* < 0.001), except at the international level, where the sample size was smaller. Boys who did not compete and those competing at the local level trained more hours per week than girls (*p* < 0.001). However, girls competing at the national level trained more hours than boys (*p* < 0.001), while no significant differences were observed between boys and girls at the regional and international levels (Table 2).

Across all athletes, physical activity levels declined with age (11–13 years: 2.96 ± 0.6; 17–19 years: 2.56 ± 0.6; *p* < 0.001), while training hours increased (11–13 years: 6.8 ± 4.7; 17–19 years: 10.7 ± 6.4; *p* < 0.001).

**Figure 1:**
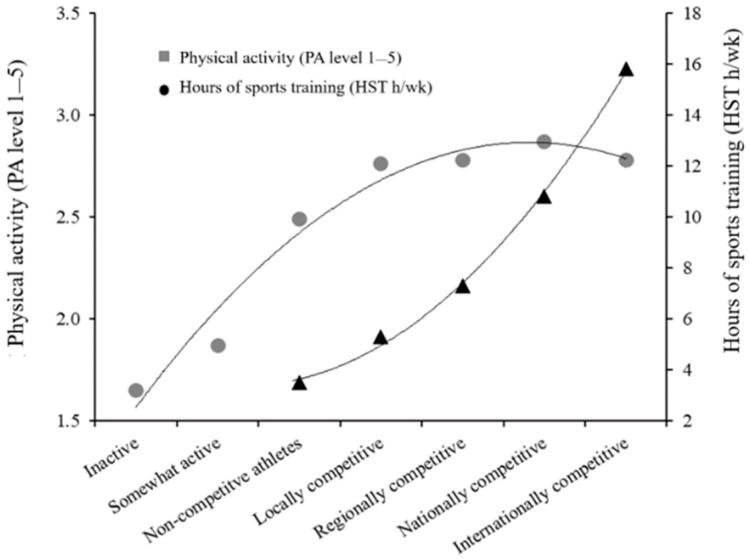
Physical activity level and hours of training per week according to competition level.

Independent of sex, athletes who competed had higher physical activity levels than non-athletes and non-competitive athletes, with only minor differences among those competing at local, regional, national, and international levels. However, competition level was strongly associated with an increase in weekly training hours (Table 2, Figure 1). A notable proportion of athletes reported physical activity levels categorized as very low to moderate (<3), including 61% of those competing at the national or international level.

We have incorporated the new, corrected figure. 

In both sexes, differences in physical activity levels by sport type were minimal. Minor differences in training hours were observed among local/regional competitors depending on the sport. Among national and international competitors, athletes in gymnastics, technical sports, endurance sports, and racket sports trained more hours per week than those in power-based, combat, or team sports (Table 3).

Athletes competing in individual sports exhibited slightly higher physical activity levels than those in team sports (2.82 ± 0.6 vs. 2.79 ± 0.6), with a notable difference in weekly training hours (10.3 ± 6.5 vs. 6.5 ± 3.7).

**Figure 2:**
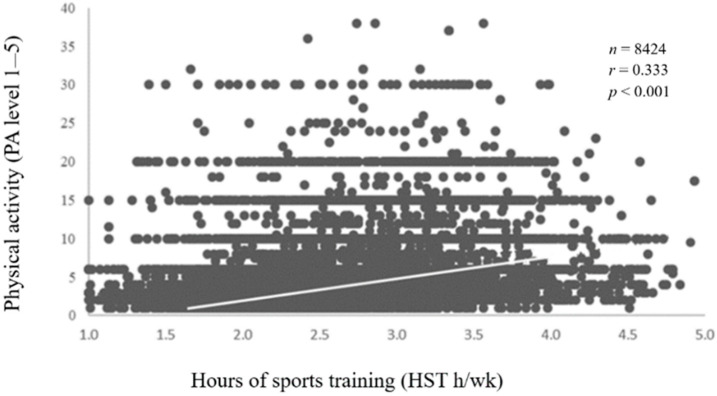
Correlation between physical activity level and weekly hours with individual data from the entire sample of athletes and trend line. Black circles represent individual athletes.

A low correlation was found between physical activity level and weekly training hours in athletes (rho = 0.333, *p* < 0.001, Figure 2), non-competitive athletes (rho = 0.271, *p* < 0.001), and competitive athletes (rho = 0.201, *p* < 0.001). Sport-specific analyses showed that most correlations were non-significant, and those that were significant ranged from low to moderate (Table 4).

## 4. Discussion

This study examined the relationship between physical activity level, assessed using the PAQ, and weekly training hours in a large sample of adolescents participating in various sports, categorized by performance level—from physically inactive individuals to international competitors. The main findings were the following: (1) girls exhibited lower physical activity levels than boys (*p* < 0.001), even at higher competition levels where both sexes had comparable training hours; (2) physical activity levels decreased with age (*p* < 0.001) despite a substantial increase in weekly training hours; (3) physical activity levels were similar across athletes competing in different sports and at different levels, despite significant differences in training hours; (4) many athletes, including those at the national and international levels, displayed low physical activity levels; and (5) no clear relationship was found between physical activity level and weekly training hours.

The results showed that boys exhibited higher physical activity levels than girls, regardless of competition level. At higher levels of competition, both male and female adolescents reported a similar number of weekly training hours. However, physical activity levels remained lower among female participants. These findings align with previous research indicating that adolescent girls tend to report lower engagement in physical activity than boys, potentially due to social, cultural, or psychological factors influencing motivation and participation in sports [24,25,26].

A notable finding was the decline in physical activity levels with increasing age, despite a significant increase in weekly training hours. This trend suggests that structured training does not necessarily translate into higher overall physical activity, a finding already reported in previous studies [27]. It is possible that as training demands increase with age and competitive level, adolescents may compensate by reducing non-structured physical activity, leading to an overall decline in their activity levels [28]. These results emphasize the importance of promoting an active lifestyle beyond structured training to counterbalance this decline.

Despite significant differences in training hours across sports and competition levels, physical activity levels remained relatively similar. Athletes involved in endurance, technical, gymnastics, and racket sports tended to train more hours per week than those in power, combat, and team sports. However, these differences in training volume did not translate into proportionally higher physical activity levels. This finding suggests that the PAQ may not fully capture the specific training demands of different sports, or that athletes in high-training-load sports engage in less non-sport-related physical activity, offsetting their total physical activity levels. This may be partially explained by the ActivityStat hypothesis, which posits that increases in structured physical activity (e.g., training) may lead to compensatory reductions in unstructured physical activity, maintaining stable overall activity levels [29]. Further, it is well known that self-reported questionnaires in general, and the PAQ in particular, are subjected to over-reporting of physical activity by less fit individuals [30]. This may have also contributed to the unexpectedly similar physical activity levels reported by both well-trained and less active participants. Given the large differences in training volume between these groups, one would expect correspondingly large differences in overall physical activity scores. This discrepancy highlights a key limitation of self-reported instruments like the PAQ, which may lack the sensitivity to distinguish between high-performance athletes and less active peers [31]. Given that the PAQ fails to adequately distinguish between athletes with different performance levels and training hours, its potential validity as a tool for the assessment of physical activity levels in adolescents could be put into question. It is possible to suggest that the PAQ may be used to investigate physical activity behaviors rather than absolute physical activity.

A notable result was the relatively low physical activity levels reported by many athletes, including those competing at the national and international levels. More than 60% of high-performance athletes reported physical activity levels categorized as low to moderate. This could be attributed to the PAQ’s focus on general activity rather than sport-specific training, potentially underestimating the activity of highly specialized athletes [12,13]. Alternatively, high-performance athletes may engage in more sedentary behaviors outside of training due to fatigue or recovery needs, contributing to lower overall activity scores [32].

Finally, there was a weak correlation between physical activity levels and weekly training hours, suggesting that increased training does not necessarily equate to higher physical activity levels. This weak association was consistent across different sports and competition levels. These results, combined with the PAQ’s limitations discussed above, highlight the setbacks of using self-reported physical activity questionnaires in athlete populations and suggest that objective measures, such as accelerometers or heart rate monitors, may provide a more accurate assessment of overall activity [33,34].

In summary, this study contributes to a better understanding of the complex relationship between sports participation, training structure, and overall physical activity in adolescents, informing future interventions aimed at promoting healthier activity patterns across diverse youth populations.

### Strengths, Limitations, and Future Research

The present study provides novel insights into the physical activity levels of adolescent athletes and practitioners, highlighting discrepancies between structured weekly training sessions and overall physical activity levels. Its strengths include a large and diverse sample (10,196 participants) spanning multiple performance levels and sport types, as well as the simultaneous analysis of physical activity and training volume across age and sex.

However, this study also presents some limitations. First, the use of a self-reported physical activity questionnaire (PAQ-C/PAQ-A) may not fully capture sport-specific or high-intensity training patterns, particularly among competitive athletes. This limits the precision of direct comparisons between training hours and global physical activity scores. Second, the cross-sectional design prevents causal interpretations. Finally, we did not collect detailed data on participants’ daily time use beyond sports (e.g., school workload, homework, employment, or other extracurricular demands). Which limits our understanding of how other life domains may affect physical activity levels during adolescence. For example, academic pressures and time allocated to school-related activities often increase during adolescence, potentially displacing opportunities for physical activity [35].

Future research should incorporate objective measurement tools (e.g., accelerometers or wearable devices) to complement self-reported data and improve the accuracy of physical activity assessments. In addition, examining how training structure, sport specialization, and recovery practices affect daily activity patterns could help inform better programming. Future studies should explore the underlying reasons for this discrepancy and consider interventions to promote higher activity levels among female athletes. Finally, time-use data would offer valuable context to understand the potential trade-offs adolescents face between sports, academics, and other responsibilities.

## 5. Conclusions

The current study provides valuable insights among adolescent practitioners and athletes, revealing discrepancies between structured weekly training sessions and overall activity levels. The findings indicate that physical activity levels decrease with age despite an increase in training hours. They also reveal that girls exhibit lower physical activity levels than boys, even when training hours are comparable. Although training hours increased with competitive level, this did not translate into proportionally higher physical activity levels. Furthermore, no strong correlation was observed between self-reported physical activity (PAQ scores) and weekly training volume, regardless of sport type or competition level.

These results suggest that structured sports participation alone is not sufficient to ensure high overall physical activity in youth populations. They also raise questions about the ability of self-reported tools like the PAQ to capture the full extent of sport-specific activity in adolescent athletes.

Future research should aim to refine physical activity assessment methods, incorporate objective measurement tools, and explore the role of training structure, recovery, and lifestyle factors that may influence daily physical activity levels, especially in girls and older adolescents.

## Figures and Tables

**Table 1:** Participant characteristics according to performance level and sex.

	Non-Athletes				Athletes									
	Inactive		Somewhat Active		Non-Competitive		Locally Competitive		Regionally Competitive		Nationally Competitive		Internationally Competitive	
	Boys	Girls	Boys	Girls	Boys	Girls	Boys	Girls	Boys	Girls	Boys	Girls	Boys	Girls
*n*	321	830	176	445	1523	2069	1558	568	499	342	976	697	100	92
Age (y)	15.6 (1.6)	15.5 (1.6)	15.5 (1.8)	15.2 (1.6)	15.1 (1.7)	14.8 (1.7)	14.6 (1.7)	14.5 (1.6)	15.0 (1.7)	14.9 (1.8)	15.5 (1.9)	15.2 (2.0)	16.9 (1.7)	16.5 (1.9)
BMI (kg/m^2^)	21.7 (3.8)	20.7 (3.6)	21.4 (4.6)	20.7 (3.5)	21.0 (3.6)	20.2 (3.9)	20.1 (3.0)	19.8 (2.9)	20.5 (3.1)	19.8 (2.7)	20.8 (2.9)	20.0 (2.9)	22.3 (2.5)	20.9 (2.8)
Sport training														
Years (y)	NA	NA	NA	NA	4.1 (3.3)	3.7 (3.1)	6.9 (3.3)	4.6 (2.9)	6.5 (3.3)	6.4 (3.3)	7.1 (3.4)	6.6 (3.2)	9.3 (3.2)	8.5 (3.3)
Days week (d/wk)	NA	NA	NA	NA	3.3 (1.6)	2.8 (1.5)	3.6 (1.2)	3.0 (1.1)	3.9 (1.1)	3.8 (1.2)	4.7 (1.3)	4.7 (1.3)	5.7 (1.2)	5.4 (1.2)

Values are the mean (standard deviation). Y, years; BMI, body mass index; NA, not applicable because these groups do not perform sports training.

**Table 2:** Physical activity levels and hours of sports training according to performance level.

	Non-Athletes				Athletes										
	Inactive		Somewhat Active		Non-Competitive		Locally Competitive		Regionally Competitive		Nationally Competitive		Internationally Competitive		*p*
	*n*	Mean (SD)	*n*	Mean (SD)	*n*	Mean (SD)	*n*	Mean (SD)	*n*	Mean (SD)	*n*	Mean (SD)	*n*	Mean (SD)	
All															
Physical activity (PA level 1–5)	1151	1.65 (0.5)	621	1.87 (0.5) *	3592	2.49 (0.6) *+	2126	2.76 (0.6) *+^	841	2.78 (0.6) *+^	1673	2.87 (0.6) *+^#	192	2.78 (0.6) *+^	<0.001
Hours of sports training (HST h/wk)		NA		NA		3.5 (2.6)		5.3 (3.0) ^		7.3 (3.9) ^#		10.8 (5.7) ^#©		15.8 (7.7) ^#©~	<0.001
Boys															
Physical activity (PA level 1–5)	321	1.75 (0.5)	176	1.99 (0.5) *	1523	2.62 (0.6) *+	1558	2.85 (0.6) *+^	499	2.83 (0.6) *+^	976	2.91 (0.6) *+^	100	2.83 (0.7) *+^	<0.001
Hours of sports training (HST h/wk)		NA		NA		4.0 (2.8)		5.6 (3.1) ^		7.3 (3.7) ^#		10.3 (5.2) ^#©		15.5 (7.4) ^#©~	<0.001
Girls															
Physical activity (PA level 1–5)	830	1.62 (0.4)	445	1.83 (0.5) *	2069	2.39 (0.6) *+	568	2.53 (0.6) *+^	342	2.71 (0.6) *+^#	697	2.82 (0.6) *+^#	92	2.74 (0.6) *+^	<0.001
Hours of sports training (HST h/wk)		NA		NA		3.2 (2.4)		4.4 (2.4) ^		7.4 (4.1) ^#		11.4 (6.2) ^#©		16.0 (8.0) ^#©~	<0.001

PA, physical activity; HST, hours of sports training. NA, not applicable because these groups do not perform sports training. Generalized linear model (GLM) for the Gamma distribution (glmGamma), factors-adjusted: socioeconomic level, maturational development, place of residence, and size of municipality. Bonferroni Post Hoc *p* < 0.05. * *p* < 0.001 vs. Inactive; + *p* < 0.001 vs. Somewhat active; ^ *p* < 0.001 vs. Non-competitive; # *p* < 0.001 vs. Locally competitive; © *p* < 0.001 vs. Regionally competitive; ~ *p* < 0.001 vs. Nationally competitive.

**Table 3:** Physical activity levels and hours of sports training according to the type of sport.

	Technical		Power		Gymnastic		Combat		Racket		Team		Endurance		*p*
	*n*	Mean (SD)	*n*	Mean (SD)	*n*	Mean (SD)	*n*	Mean (SD)	*n*	Mean (SD)	*n*	Mean (SD)	*n*	Mean (SD)	
All															
Physical activity (PA level 1–5)	165	2.85 (0.7)	127	2.79 (0.5)	350	2.70 (0.6)	214	2.81 (0.6)	227	2.84 (0.6)	2908	2.80 (0.6)	781	2.87 (0.6) ^	<0.005
Hours of sports training (HST h/wk)		12.1 (7.8)		8.9 (4.1) *		11.5 (7.9) +		8.4 (5.4) *^		6.9 (5.4) *+^#		6.5 (3.7) *+^ #		10.9(6.0) +#©~	<0.001
Locally/regionally competitive															
Physical activity (PA level 1–5)	35	2.73 (0.8)	23	2.69 (0.4)	180	2.56 (0.6)	69	2.79 (0.6)	160	2.78 (0.6) ^	2230	2.78 (0.6) ^	255	2.80 (0.6) ^	<0.001
Hours of sports training (HST h/wk)		5.9 (3.7)		5.4 (2.6)		6.4 (4.2)		5.6 (4.1)		5.0 (3.2) ^		5.7 (3.0) ©		7.8 (5.0) *+^#©~	<0.001
Nationally/internationally competitive															
Physical activity (PA level 1–5)	130	2.88 (0.6)	104	2.81 (0.5)	170	2.87 (0.5)	145	2.83 (0.7)	67	3.00 (0.6)	678	2.84 (0.6)	526	2.90 (0.6)	0.334
Hours of sports training (HST h/wk)		13.8 (7.8)		9.7 (3.9) *		16.9 (7.2) *+		9.7 (5.5) *^		11.5 (6.6) ^		9.2 (4.4) *^©		12.4 (5.9) +^# ~	<0.001

PA, physical activity; HST, hours of sports training. Generalized linear model (GLM) for the Gamma distribution (glmGamma), factors-adjusted: socioeconomic level, maturational development, place of residence, and size of municipality. Bonferroni Post Hoc *p* < 0.05. * *p* < 0.001 vs. Technical; + *p* < 0.001 vs. Power; ^ *p* < 0.001 vs. Gymnastic; # *p* < 0.001 vs. Combat; © *p* < 0.001 vs. Racket; ~ *p* < 0.001 vs. Team.

**Table 4:** Correlation between physical activity level and training hours according to the type of sport.

	**All**		**Boys**		**Girls**	
	**n**	**rho**	**n**	**rho**	**n**	**rho**
Technical sports	165	0.156 *	79	−0.118	86	0.341 *
Power sports	127	0.060	68	0.207	59	−0.116
Gymnastic sports	350	0.390 *	23	0.128	327	0.399 *
Combat sports	214	0.284 *	139	0.179 *	75	0.488 *
Racquet sports	227	0.208 *	138	0.116	89	0.277 *
Team sports	2908	0.181 *	2224	0.149 *	684	0.251 *
Endurance sports	704	0.185	398	0.136 *	306	0.225 *
Uncategorized sports	47	−0.135	28	−0.035	19	−0.398

Correlations were calculated using Spearman’s correlation coefficient. * *p* < 0.05.

## Data Availability

No data are available at this stage, as this is a study protocol. Data will be collected during the study and, upon completion, will be made available in a publicly accessible repository, subject to privacy and ethical considerations.
